# Supplementary material for: The Role of miRNAs to Detect Progression, Stratify, and Predict Relevant Clinical Outcomes in Bladder Cancer
Source: Int J Mol Sci. 2024 Feb 11;25(4):2178. doi: 10.3390/ijms25042178 (PMC10889402; doi:10.3390/ijms25042178)
Supplement: Supplementary file 1 [file ijms-25-02178-s001.zip › ijms-2804438-supplementary.pdf]

Supplementary Table S1. Tumor, Node, Metastasis Classification (TNM) Classification of BC.

2004 WHO/ISUP	Pathways	TNM classification		
**Primary tumor (T)**				
		Tx		Primary tumors cannot be assessed
		T0		No evidence of a primary tumor
Papillary urothelial neoplasm of low malignant potential (PUNLMP)	NMIBC	Tis		Carcinoma in situ is a flat, poorly differentiated tumor confined to mucosa
Low grade		Ta		Non-invasive papillary carcinoma, confined to the mucosa
		T1		Tumor invades subepithelial connective tissue (lamina propria)
	MIBC	T2<br>Tumor invades muscle	T2a	Tumor invades superficial muscularis propria (inner half)
High grade			T2b	Tumor invades deep muscularis propria (outer half)
		T3<br>Tumor invades perivesical tissue	T3a	Tumor invades perivesical fat microscopically
			T3b	Tumor invades perivesical fat macroscopically
		T4<br>Tumor invades any of the following: prostate stroma, seminal vesicles, uterus, vagina pelvic wall, abdominal wall	T4a	Tumor invades prostate stroma, seminal vesicles, uterus, or vagina
			T4b	Tumor invades pelvic wall or abdominal wall
**Regional lymph nodes (N)**				
		NX		Lymph nodes cannot be assessed
		N0		No lymph node metastasis
		N1		Single regional lymph node metastasis in the true pelvis (hypogastric, obturator, external iliac or presacral lymph node)
		N2		Multiple regional lymph node metastasis in the true pelvis (hypogastric, obturator, external iliac, or presacral lymph node)
		N3		Metastasis in common iliac lymph node(s)
**Distant metastasis (M)**				
		M0		No distant metastasis
		M1		Distant metastasis
